# LHX2: a transcription factor in development, homeostasis, repair, and disease

**DOI:** 10.3389/fcell.2026.1835210

**Published:** 2026-06-12

**Authors:** Pengfei Yang, Jinpeng Hu, Xinqiao Li, Zinan You, Jinpeng Zhou, Zhitao Jing

**Affiliations:** 1 Department of Neurosurgery, The First Hospital of China Medical University, Shenyang, Liaoning, China; 2 Department of Neurosurgery, Liaoning Cancer Hospital and Institute, Cancer Hospital of China Medical University, Shenyang, Liaoning, China; 3 Department of Neurosurgery, Tangdu Hospital, Fourth Military Medical University, Xi’an, Shaanxi, China

**Keywords:** Lhx2, organogenesis, regenerative medicine, tissue homeostasis, transcriptional regulation

## Abstract

LIM homeobox 2 (LHX2) is a LIM-homeodomain (HD) transcription factor that participates in regulating vertebrate development, tissue homeostasis, and injury repair. This review systematically synthesizes the pleiotropic functions of LHX2 across multiple organ systems. During development, LHX2 directs regional specification and cell fate determination in the cerebral cortex, hippocampus, and retina, while regulating limb bud patterning and the development of the visual, olfactory, reproductive, and hematopoietic systems. During homeostasis, LHX2 acts as a key negative regulator to maintain the quiescent state of hepatic stellate cells (HSCs), osteoclast precursors, astrocytes, and hair follicle stem cells, thereby preserving tissue integrity. Following injury, LHX2 switches to a pro-regenerative mode, promoting re-epithelialization, axon regeneration, and hematopoietic niche remodeling. Dysregulation of LHX2 is causally linked to neurodevelopmental disorders, ocular malformations, liver fibrosis, osteoporosis, and various cancers. Emerging evidence also implicates LHX2 in metabolic-epigenetic circuits, the phosphoinositide 3-kinase (PI3K)/protein kinase B (AKT)/mechanistic target of rapamycin (mTOR) and Wingless/Int-1 (Wnt)/β-catenin axes, as well as microRNA (miRNA) regulation. We also discuss therapeutic strategies targeting LHX2, including molecular engineering to overcome species barriers, and outline key knowledge gaps. A comprehensive understanding of LHX2-regulated networks holds promise for advancing regenerative medicine and enabling precision interventions in developmental disorders.

## 1 Introduction

**FIGURE 1:**
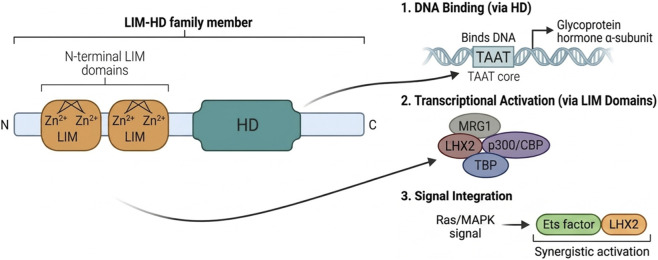
Structure and function of the LHX2 protein. The LHX2 protein comprises two N-terminal LIM domains (each containing two Zn^2+^-coordinated zinc finger motifs) and a C-terminal homeodomain (HD). The HD recognizes the TAAT core motif within target gene promoters (e.g., the glycoprotein hormone alpha subunit promoter), enabling direct DNA binding. The LIM domains act as transcriptional activation modules: they recruit coactivators (e.g., MSG1-related gene 1 (MRG1)) and general transcription machinery components (e.g., TATA-box binding protein (TBP), E1A binding protein p300 (p300)/CREB-binding protein (CBP)) via protein-protein interactions, thereby assembling active transcriptional complexes and promoting basal transcription. Additionally, LHX2 cooperates with signaling pathway effectors (e.g., ETS factors activated by the rat sarcoma (Ras)/ mitogen-activated protein kinase (MAPK) cascade) to mediate synergistic transcriptional responses.

LIM homeobox 2 (LHX2) is a LIM-homeodomain (HD) protein encoded by the *LHX* gene family (Singh et al., 2022a; Yasuoka and Taira, 2021; Zhang et al., 2023), and was first identified in a pre-B cell line (Xu et al., 1993; Roberson et al., 1994) (Figure 1). LHX2 is recognized as a central orchestrator of diverse biological processes, ranging from early embryonic patterning to adult tissue homeostasis and injury repair (Chou and Tole, 2019; Porter et al., 1997; Hobert and Westphal, 2000). It integrates multiple extracellular signals (e.g., bone morphogenetic protein (BMP), fibroblast growth factor (FGF), Wingless/Int-1 (Wnt), nuclear factor κ-light-chain-enhancer of activated B Cells (NF-κB), transforming growth factor-β (TGF-β)) in a context-dependent manner to orchestrate cell-type-specific transcriptional programs. Through chromatin-based regulation, phase separation, and context-dependent signal integration, LHX2 achieves its functional versatility, thereby playing essential roles in guiding cell fate, maintaining quiescence, and promoting regeneration (Glenn and Maurer, 1999; Britton et al., 2025; Li et al., 2022a; Muralidharan et al., 2017a; Monahan et al., 2019).

Extensive studies have demonstrated that LHX2 is broadly involved in the development and homeostasis of multiple organ systems (Singh et al., 2022a; Yasuoka and Taira, 2021; Porter et al., 1997), including the nervous system (cortical regionalization (Mangale et al., 2008; Chou et al., 2009)), visual system (optic cup formation (Yun et al., 2009)), olfactory system (olfactory neuron differentiation (Monahan et al., 2019; Hirota and Mombaerts, 2004; Kolterud et al., 2004a)), digestive system (hepatic stellate cell (HSC) quiescence (Wandzioch et al., 2004; Kolterud et al., 2004b)), reproductive system (ovarian vascular patterning (Singh et al., 2022b; Singh et al., 2023)), hematopoietic system (hematopoietic stem cell maintenance (Pinto Do et al., 2002; Pinto Do et al., 2001; Richter et al., 2003)), musculoskeletal system (limb patterning (Britton et al., 2025; Watson et al., 2018), bone remodeling balance (Kim et al., 2014)) and the skin and its appendages (skin wound healing (Mardaryev et al., 2011)). Dysregulation of LHX2 has also been causally linked to neurodevelopmental disorders (Porter et al., 1997; Schmid et al., 2023), ocular malformations (Porter et al., 1997; Yun et al., 2009; Tétreault et al., 2009), liver fibrosis (Wandzioch et al., 2004; Kolterud et al., 2004b), osteoporosis (Kim et al., 2014), and various cancers (Ohata et al., 2025). In addition to its roles in development and homeostasis, LHX2 is also activated upon injury to promote tissue repair, including axon regeneration and wound re-epithelialization (Mardaryev et al., 2011; Li et al., 2024).

Despite considerable progress, a unified framework that integrates the molecular mechanisms, context-dependent functions, and therapeutic potential of LHX2 is still lacking (Muralidharan et al., 2017a; Monahan et al., 2019). Therefore, this review systematically consolidates current knowledge on LHX2, with a focus on its structural basis, cell-type-specific roles in development and disease, and emerging strategies to target LHX2-regulated networks. Our aim is to bridge fundamental biology with clinical practice and to promote further research into LHX2 as a critical node in development, homeostasis, and repair.

## 2 LHX2 in development

LHX2 functions as a multifunctional transcriptional regulatory hub that integrates upstream signals and precisely controls gene expression (Glenn and Maurer, 1999). During development, LHX2 guides cell fate, maintains progenitor proliferation, and coordinates organ morphogenesis.

### 2.1 Nervous system

During early embryogenesis, LHX2 acts as a repressor of secondary organizers and a selector gene for forebrain development (Chou and Tole, 2019; Porter et al., 1997; Mangale et al., 2008). In cortical regionalization, it represses hippocampal, antihem, and paleocortex programs, directing the dorsal telencephalon to a neocortical fate, and similarly suppresses hem fate in the hippocampal primordium (Bulchand et al., 2001; Monuki et al., 2001; Vyas et al., 2003; Muzio and Mallamaci, 2005; Godbole et al., 2018). Ablation before embryonic day (E)10.5 causes neocortical progenitors to switch fate (Mangale et al., 2008; Chou et al., 2009). In addition to its role in regional specification, LHX2 maintains cortical progenitor proliferation by regulating a paired box 6 (*PAX6*) enhancer and driving hes family bHLH transcription factor 1 (*HES1*) expression (a notch homolog protein (Notch) effector). Conditional knockout leads to premature cell cycle exit, accelerated differentiation, and reduced cortical volume (Chou and O’leary, 2013; Shetty et al., 2013; Hsu et al., 2015; Srivastava et al., 2025). After neuronal identity is established, LHX2 exerts opposing effects on deep-versus upper-layer neurons. In deep-layer neurons, it recruits the lysine-specific demethylase 1 (LSD1)/histone deacetylase 2 (HDAC2)/ retinoblastoma-binding protein 4 (RBBP4) complex to elevate histone modification histone H3 lysine nine dimethylation (H3K9me2), silencing FEZ family zinc finger 2 (*FEZF2*) and SRY-box transcription factor 11 (*SOX11*). In contrast, it transactivates the Cut-like homeobox 2 (*CUX2*)-E1 enhancer to promote *CUX2* expression in upper-layer neurons (Muralidharan et al., 2017a; Yang H. et al., 2020).

Beyond cortical patterning, LHX2 controls subplate neuron development, regulating the waiting period of thalamocortical axons. Its loss causes premature axonal invasion and degeneration of sensory thalamic neurons. LHX2 also drives barrel formation independently of neural activity, acts as a terminal selector for sensory areal identity, and regulates dendritic arborization of callosal projection neurons (Shetty et al., 2013; Pal et al., 2021; Wang et al., 2017; Zembrzycki et al., 2015; Wang et al., 2023).

**FIGURE 2:**
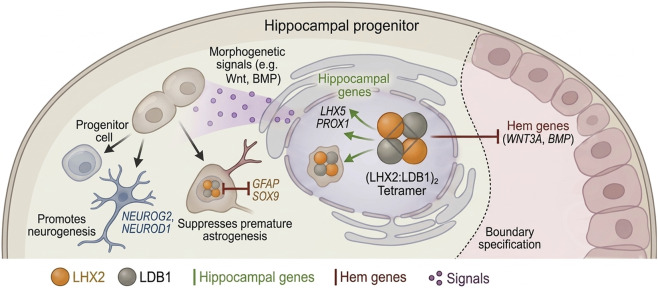
Role of the LHX2:LDB1 tetrameric complex in hippocampal primordium patterning and neural progenitor fate determination. LHX2 (orange) forms a stable tetrameric complex with LDB1 (grey) within the nucleus of hippocampal progenitors. This complex drives hippocampal regional specification by activating hippocampal fate genes (e.g., *LHX5*, Prospero homeobox 1 (*PROX1*)) and repressing cortical hem identity genes (e.g., Wnt family member 3A (*WNT3A*), *BMP*), thereby establishing a boundary between the hippocampal primordium and the adjacent cortical hem. The complex also regulates the neuron-glia fate switch and maintains neurogenic potential. Morphogenetic signals (e.g., Wnt, BMP) from the cortical hem modulate this activity.

During hippocampal development, LHX2 promotes neurogenesis and inhibits premature astrogliogenesis, shaping the hippocampus-specific chromatin landscape (Kinare et al., 2020; Suresh et al., 2023) (Figure 2). It cross-regulates with doublesex-and mab-3-related transcription factor A2 (DMRTA2) to control the neuron-glia fate switch (Subramanian et al., 2011; Muralidharan et al., 2017b). In other brain regions, combined loss of *LHX2* and *LHX9* in the thalamus blocks neuronal differentiation and permits ectopic Wnt-expressing tissue, disrupting anterior-posterior patterning (Peukert et al., 2011). Pituitary development is also affected. *LHX2* deletion compromises infundibulum formation and alters lobe structure (Zhao et al., 2010). Additionally, LHX2 regulates neuronal migration and glial wedge progenitors during corpus callosum development. Its deletion causes agenesis, impairing interhemispheric communication (Yang et al., 2024; Chinn et al., 2015).

Collectively, these findings show that LHX2 operates as a master regulator across forebrain regions, employing region-specific partnerships and chromatin mechanism.

### 2.2 Visual system

LHX2 is a key member of the eye field transcription factor network, which orchestrates vertebrate eye development from the earliest stages (Fitzpatrick and Van Heyningen, 2005; Hägglund et al., 2011; Zuber et al., 2003). At the anterior neural plate stage, LHX2 is required for eye field specification and directly activates downstream transcription factors including retina and anterior neural fold homeobox (RAX), PAX6, and SIX homeobox 3 (SIX3), each of which further promotes regionalization and proliferation of the optic primordium (Hägglund et al., 2011; Zuber et al., 2003). Loss of *LHX2* arrests optic vesicle development at an early stage, thereby preventing subsequent optic cup formation and lens induction (Tétreault et al., 2009).

**FIGURE 3:**
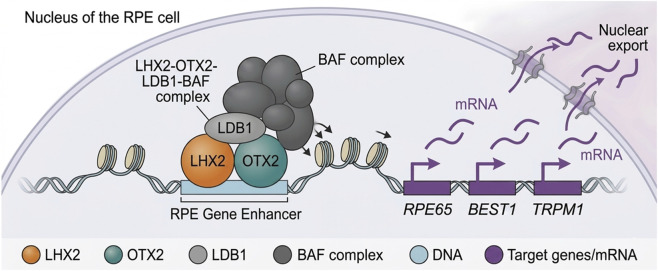
Synergistic actions of LHX2 with its cofactors in regulating gene expression within the nucleus of RPE cells. LHX2 (orange) forms a multiprotein complex with OTX2 (teal), LDB1 (grey), and the BRG1/BRM-associated factor (BAF) chromatin remodeling complex (dark grey). This LHX2-OTX2-LDB1-BAF complex is recruited to enhancer regions of RPE-specific genes. The complex recruits the BAF chromatin remodeling complex, which remodels chromatin into an open conformation, thereby allowing access to the transcriptional machinery. This cooperative activity drives the expression of key RPE functional genes, including RPE-specific 65 kDa (*RPE65*), bestrophin 1 (*BEST1*), and transient receptor potential cation channel subfamily M member 1 (*TRPM1*). The resulting mRNA is subsequently exported from the nucleus to support the specialized functions of the RPE. This model highlights the core role of LHX2 as a synergistic transcriptional regulator that collaborates with tissue-specific factors (e.g., OTX2) and chromatin remodelers to control terminal differentiation and function of the RPE.

During the optic vesicle-to-cup transition, LHX2 acts as a mediator of BMP4/7 autocrine and paracrine signaling. By transducing these extracellular cues, it coordinates the dynamic remodeling of the optic vesicle into the bilayered optic cup while simultaneously promoting lens placode development (Yun et al., 2009; Roy et al., 2013). At later stages, LHX2 synergizes with orthodenticle homeobox 2 (OTX2) to regulate retinal pigment epithelium (RPE)-specific gene expression, a partnership essential for terminal RPE differentiation and function (Cohen-Gulkar et al., 2023) (Figure 3).

In the lens, LHX2 promotes fiber cell proliferation, survival, and differentiation by regulating the expression of fibroblast growth factor (*FGF*) derived from the neural retina, thereby linking neural retina-derived signals to lens morphogenesis (Thein et al., 2016). Within retinal progenitor cells (RPCs), LHX2 is persistently expressed to regulate the timing of cell cycle exit, ensuring a proper balance between progenitor maintenance and neuronal output (Thein et al., 2016; Gordon et al., 2013). Furthermore, LHX2 is indispensable for retinal Müller glia differentiation. It activates Notch pathway components and gliogenic factors (e.g., hes family bHLH transcription factor 5 (HES5), SRY-box transcription factor 8 (SOX8)), thereby directing glial specification and terminal maturation while dynamically balancing retinal neurogenesis and gliogenesis (De Melo et al., 2016a; De Melo et al., 2016b; De Melo et al., 2018).

### 2.3 Olfactory system

LHX2 acts as a master upstream determinant for odorant receptor (OR) choice, binding to conserved elements within OR promoters or enhancers to initiate monoallelic OR expression. Its deletion abolishes nearly all tested OR genes, particularly class II and a subset of class I ORs (Hirota and Mombaerts, 2004; Hirota et al., 2007). As a core component of the LHX2/early B-cell factor (EBF)/ LIM domain binding protein 1 (LDB1) complex, LHX2 assembles multi-chromosomal transcriptional hubs via phase separation, silencing competing OR alleles while recruiting the chosen allele into the active hub, thereby ensuring stable expression of a single OR (Monahan et al., 2019; Zhang et al., 2016).

During olfactory sensory neuron (OSN) differentiation, LHX2 enables progenitors to generate mature OR-expressing OSNs. Loss of *LHX2* arrests OSN maturation at the OR choice stage, without affecting early progenitor regionalization or pan-neuronal identity (Kolterud et al., 2004a). In OSNs, LHX2 also autonomously controls axon specification and targeting (Berghard et al., 2012).

In the olfactory bulb, *LHX2* mutation causes structural abnormalities and severe defects in the lateral olfactory tract (LOT) projection path, associated with mispositioning of guidepost cells (e.g., LOT cells) and aberrant semaphorin 6 A (SEMA6A) expression (Saha et al., 2007). Through OSN-derived axonal inputs, LHX2 non-autonomously influences olfactory bulb lamination, vomeronasal nerve development, and indirectly guides gonadotropin-releasing hormone (GnRH) neuron migration (Berghard et al., 2012).

### 2.4 Reproductive system

LHX2 exhibits sexually dimorphic expression in fetal germ cells. It serves as a key early determinant of gonadal vascular patterning. As a core transcription factor, it enables germ cells to actively construct their own microenvironment, thereby coordinating gonadal morphogenesis and functional differentiation (Singh et al., 2022b; Singh et al., 2023). In the testis, LHX2 is specifically expressed in fetal germ cells, where it directly regulates germ cell fate while indirectly influencing Sertoli, Leydig, and endothelial cell gene expression as a local signaling center. Loss of *LHX2* leads to aberrant endothelial migration, Leydig cell hyperplasia, and severe disruption of testis cord organization, underscoring its indispensable role in maintaining the normal testicular microenvironment (Singh et al., 2023). In the ovary, during the critical window of sex determination, LHX2 shows a conserved female-biased expression pattern. Through transcriptional repression of germ cell-derived pro-angiogenic signals, LHX2 non-cell-autonomously shapes the ovarian microenvironment, actively suppressing endothelial cell migration and excessive vascularization to maintain the characteristic hypovascular state. Deletion of *LHX2* disrupts this balance, causing ectopic endothelial migration and a testis-like vascular architecture, further confirming its critical role in preventing excessive ovarian vascularization (Singh et al., 2022b). Thus, LHX2 coordinates gonadal vascular patterning in a sexually dimorphic manner, suppressing angiogenesis in the ovary while promoting testis cord organization.

### 2.5 Musculoskeletal system

During limb development, LHX2 serves as a critical mediator that converts FGF signals from the apical ectodermal ridge (AER) into sonic hedgehog (*SHH*) expression in the distal mesenchyme, thereby linking limb outgrowth to anteroposterior patterning. LHX2 is rapidly induced as a direct transcriptional target of AER-derived FGF signals and becomes precisely restricted to the zone of polarizing activity (ZPA), a key signaling center that specifies digit identity. Concurrently, LHX2 sustains the expression of *FGF10* to support AER integrity, proximodistal elongation, and proper anteroposterior patterning. Disruption of LHX2 function perturbs this integrated signaling circuitry, ultimately leading to severe limb truncation (Britton et al., 2025; Li et al., 2022a; Watson et al., 2018).

The expression of *LHX2* itself is tightly controlled by two limb-specific cis-regulatory modules, LHX2-associated distal limb regulatory module (LADLRM) and LHX2-associated sub-AER regulatory module (LASARM), which integrate inputs from E26 transformation-specific (ETS) transcription factors downstream of FGF signaling and T-cell factor (TCF)/lymphoid enhancer factor (LEF) factors downstream of Wnt/β-catenin signaling. These modules ensure that *LHX2* is expressed in the correct spatial domain and at the appropriate levels during limb bud outgrowth (Britton et al., 2025). Of note, dorsoventral patterning of the limb is governed by separate factors such as LIM homeobox transcription factor 1 (LMX1), indicating that during evolution, the genetic programs controlling limb growth and specification have been decoupled into independent modules, each regulated by its own set of signaling inputs (Rodriguez-Esteban et al., 1998).

### 2.6 Hematopoietic system

Lhx2 is indispensable for normal embryonic hematopoiesis. Its loss results in severe anemia and intrauterine death in mouse embryos, underscoring its essential role in terminal erythroid production and overall hematopoietic development (Porter et al., 1997). *In vitro* studies using mouse embryonic stem cell-derived embryoid bodies have demonstrated that Lhx2 induces self-renewal of multipotent hematopoietic progenitors, maintaining their stemness while promoting multilineage differentiation toward both erythroid and myeloid lineages (Dahl et al., 2008). *In vivo* transplantation experiments further confirm that *Lhx2*-expressing hematopoietic progenitor cells can reconstitute long-term, multilineage hematopoiesis in recipient mice (Pinto Do et al., 2002; Pinto Do et al., 2001; Richter et al., 2003). In the adult, Lhx2 contributes to the maintenance of hematopoietic stem cells through a cell-nonautonomous mechanism. Lhx2 remodels the bone marrow niche by enhancing the homing and retention of hematopoietic progenitors, thereby sustaining long-term hematopoietic output and preserving niche integrity (Pinto Do et al., 2002; Pinto Do et al., 2001).

### 2.7 Skin and its appendages

*Lhx2* expression is dynamic during mouse skin development, barely detectable at embryonic day (E) 13 prior to hair follicle formation, then becoming highly expressed in the epidermal basal layer at E15 and E17 (Takaya et al., 2022), and ultimately confined to hair placodes while remaining absent from sweat gland placodes. This spatiotemporal pattern suggests that Lhx2 acts as an early molecular switch distinguishing hair follicle from sweat gland fate (Mardaryev et al., 2011; Takaya et al., 2022; Folguera et al., 2013). In postnatal telogen follicles, *Lhx2* is expressed in the bulge region and secondary hair germ, where it colocalizes with key stem cell markers such as SRY-box transcription factor 9 (Sox9) and T-cell factor 4 (Tcf4). Within these compartments, Lhx2 is essential for maintaining stem cell quiescence and preserving hair follicle architecture. Mechanistically, it directly represses leucine-rich repeat-containing G-protein coupled receptor 5 (*Lgr5*) in the secondary hair germ to prevent premature hair cycle activation, while positively regulating *Sox9* and *Tcf4* in the bulge to uphold stem cell identity. Loss of *Lhx2* leads to progressive disintegration of the stem cell niche, aberrant cell polarization, and even fate conversion toward a sebaceous gland lineage, highlighting its role as a gatekeeper of stem cell fate (Mardaryev et al., 2011; Folguera et al., 2013). Furthermore, *Lhx2* is cyclically expressed in progenitors located outside the bulge region during the hair cycle, where it acts as a positive regulator to promote the initiation and progression of anagen entry, thereby coordinating the timely transition between quiescence and regeneration (Bohr et al., 2013). In human hair follicles, *LHX2* expression exhibits a broader distribution, extending throughout the outer root sheath from the sub-bulge region to the proximal bulb. This expanded expression domain suggests that LHX2 may define and regulate a distinct progenitor subset in humans, contributing to the establishment and maintenance of follicular epithelial cellularity and homeostasis beyond the conventional stem cell niche (Purba et al., 2015).

## 3 LHX2 in tissue homeostasis

In addition to its functions during development, LIM homeobox 2 (LHX2) also plays critical roles in the maintenance of tissue homeostasis across multiple organ systems, primarily by preserving cellular quiescence and preventing aberrant activation.

### 3.1 Nervous system

LHX2 exerts an indispensable dual regulatory role in maintaining neural cell homeostasis, covering both astrocytes and neurons. In astrocytes, *LHX2* is persistently expressed in glial progenitors within the subventricular zone and in mature astrocytes, where it actively suppresses aberrant proliferation and activation. Mechanistically, LHX2 directly represses proliferation-associated genes and maintains the quiescent state of astrocytes by antagonizing pro-inflammatory signaling pathways. Conditional knockout of *Lhx2* in neonatal mice leads to selective hyperproliferation of upper-layer cortical astrocytes and spontaneous activation of reactive gliosis programs, as evidenced by marked upregulation of glial fibrillary acidic protein (GFAP), thereby disrupting the stability of the neural microenvironment (Iyer et al., 2025). In neurons, Lhx2 preserves the structural and functional integrity of mature upper-layer cortical neurons. It also prevents aberrant neuronal hyperexcitability, thereby contributing to neuronal functional integrity. Through this multi-level regulation, simultaneously inhibiting astrocyte reactive activation and sustaining neuronal functional homeostasis, Lhx2 emerges as a core transcription factor that supports overall neural cell homeostasis and microenvironmental stability, linking glial and neuronal compartments in a coordinated regulatory network (Yang H. et al., 2020; Iyer et al., 2025).

### 3.2 Digestive system

*LHX2* is specifically expressed in hepatic stellate cells (HSCs) and acts as a core regulator that prevents liver fibrosis and ensures overall liver homeostasis. Under normal physiological conditions, LHX2 strictly keeps HSCs quiescent by directly repressing the expression of activation-associated genes and maintaining the cells in a non-fibrogenic state. This effectively inhibits their transdifferentiation into myofibroblasts, thereby preventing aberrant deposition of extracellular matrix components such as type I and type III collagen. *LHX2* deficiency causes spontaneous HSC activation, as evidenced by a significant increase in α-smooth muscle actin (α-SMA)-positive cells and upregulation of profibrotic factors including platelet-derived growth factor (PDGF) and matrix metalloproteinases (MMPs), ultimately resulting in progressive liver fibrosis and disruption of hepatic tissue architecture. Transfection experiments have validated this inhibitory effect. Introduction of *LHX2* complementary DNA (cDNA) into a human HSC line significantly downregulates activation-associated genes, confirming that LHX2 is both necessary and sufficient to maintain HSC quiescence (Wandzioch et al., 2004; Kolterud et al., 2004b). Mechanistically, LHX2 exerts a coordinated dual transcriptional activity. On one hand, it transcriptionally activates SMAD family member 6 (*SMAD6*), which antagonizes transforming growth factor-β (TGF-β) signaling, thereby blocking HSC activation, proliferation, and extracellular matrix synthesis. On the other hand, LHX2 upregulates Hepatocyte growth factor (*HGF*) expression, which directly stimulates hepatocyte proliferation and promotes recovery of impaired liver function. This balanced regulation, suppressing fibrotic pathways while supporting parenchymal regeneration, positions LHX2 as a master homeostatic guardian in the liver (Wandzioch et al., 2004).

### 3.3 Musculoskeletal system

In the precise dynamic balance between osteoblast-mediated bone formation and osteoclast-mediated bone resorption, LHX2 acts as a negative regulator that limits osteoclast generation. Through its N-terminal tandem LIM domains, LHX2 directly binds to the cellular Fos proto-oncogene (c-Fos) protein, physically blocking c-Fos from binding to the nuclear factor of activated T Cells cytoplasmic 1 (*NFATC1*) promoter. Since NFATC1 is the master transcription factor for osteoclast differentiation, this interaction effectively prevents the initiation of the osteoclast differentiation program and maintains bone resorption at physiological levels (Kim et al., 2014). Under normal conditions, receptor activator of nuclear factor κ-B ligand (RANKL) signaling downregulates *LHX2* expression during the early stages of osteoclast differentiation, thereby releasing this brake on c-Fos and allowing the differentiation program to proceed as needed for normal bone remodeling. *In vivo* experiments confirm that *LHX2* conditional knockout results in a significant increase in osteoclast generation and a relative enhancement of bone resorption activity, thereby disrupting the original balance between bone formation and resorption and leading to net bone loss (Kim et al., 2014). (The role of pro-inflammatory cytokines such as tumor necrosis factor-α (TNF-α) in downregulating *LHX2* is discussed in Section 5.1.4).

### 3.4 Hematopoietic system

LHX2 plays multiple critical roles in maintaining hematopoietic homeostasis. It remodels the hematopoietic microenvironment through a cell-nonautonomous mechanism and contributes to the long-term maintenance of hematopoietic stem/progenitor cells (HSPCs). Studies have shown that Lhx2 confers sustained self-renewal capacity upon adult mouse HSPCs, enabling them to maintain long-term, multilineage hematopoietic output under physiological conditions without exhausting the stem cell pool (Pinto Do et al., 2002; Pinto Do et al., 2001). This process depends critically on a cell-nonautonomous mechanism. Lhx2 enhances the homing and retention of hematopoietic cells by shaping the bone marrow niche. Specifically, *Lhx2*-expressing cells modulate the expression of niche factors such as KIT ligand (KITLG) and chemokines, which promote HSPC adhesion, survival, and quiescence. By remodeling the microenvironment, Lhx2 ensures a balanced production of erythroid, myeloid, and lymphoid lineages over extended periods, thereby preserving hematopoietic reserve and preventing premature stem cell exhaustion (Pinto Do et al., 2002; Pinto Do et al., 2001). (Species-specific differences between mouse and human LHX2 activity are addressed in the Discussion, Section 6).

## 4 LHX2 in repair and regeneration

In addition to maintaining tissue homeostasis under physiological conditions, LIM homeobox 2 (*LHX2*) is upregulated after tissue injury and actively participates in repair and regeneration processes.

### 4.1 Nervous system

In the adult central nervous system, LHX2 significantly promotes axonal regeneration and functional recovery after injury. Using the optic nerve injury model, LHX2 directly transcriptionally represses the expression of the repulsive guidance molecule semaphorin 3C (*SEMA3C*), a potent chemorepellent that normally limits axonal outgrowth. By downregulating SEMA3C, LHX2 relieves the chemical repulsion exerted on axonal growth cones, thereby removing spatial barriers to distal axon extension and enabling regenerating axons to traverse the injury site. At the same time, LHX2 effectively enhances the intrinsic survival capacity of retinal ganglion cells (RGCs). It suppresses injury-induced apoptotic pathways and promotes the expression of neurotrophic factors, thereby reducing RGC death following optic nerve damage. These two actions, repression of extrinsic axonal repellents and enhancement of intrinsic neuronal survival, act synergistically at the injury site to collectively establish a microenvironment conducive to both axon regeneration and long-term neuronal preservation (Li et al., 2024). Thus, LHX2 promotes axon regeneration and neuroprotection, making it a promising molecular target for treating central nervous system injuries such as optic neuropathies.

### 4.2 Skin and its appendages

During mouse skin wound repair, Lhx2 acts as a core regulator of epithelial stem cell activity. Following injury, *Lhx2* expression is rapidly upregulated in migrating epidermal keratinocytes at the wound edge, where it drives cell proliferation and migration to restore the epidermal barrier. Heterozygous knockout of *Lhx2* delays re-epithelialization but unexpectedly accelerates anagen initiation in follicles adjacent to the wound, revealing a dual function: promoting wound closure while restraining premature hair cycle activation (Mardaryev et al., 2011).

**FIGURE 4:**
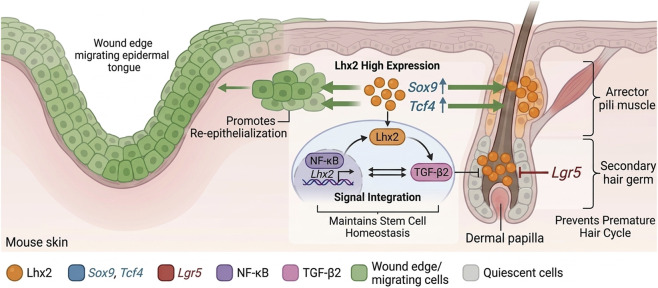
Role of Lhx2 in mouse skin wound repair and hair follicle regulation. At the wound edge, high expression of *Lhx2* in migrating epidermal keratinocytes promotes re-epithelialization by driving keratinocyte migration and proliferation. Within the hair follicle, Lhx2 acts as a core regulator of epithelial stem cell fate: it positively regulates the expression of the stem cell maintenance genes *Sox9* and *Tcf4* in the bulge region to preserve stem cell identity, while directly repressing *Lgr5* in the secondary hair germ to prevent premature hair cycle activation and anagen initiation. Mechanistically, Lhx2 integrates upstream signals from the NF-κB and TGF-β2 pathways, establishing a balanced regulatory network that maintains stem cell homeostasis and prevents niche dysregulation.

Mechanistically, Lhx2 exerts its functions by regulating the same targets described in Section 2.7 (SRY-box transcription factor 9 (*Sox9*), T-cell factor 4 (*Tcf4*), and leucine-rich repeat-containing G-protein coupled receptor 5 (*Lgr5*)). In bulge stem cells, Lhx2 activates *Sox9* and *Tcf4* to preserve stem cell identity and sustain the proliferative capacity needed for re-epithelialization. In the secondary hair germ, it represses *Lgr5*, thereby preventing premature entry into anagen that would otherwise divert cellular energy from epidermal repair. In addition to these cell-autonomous mechanisms, Lhx2 integrates intercellular signals by coordinating crosstalk between the nuclear factor κ-light-chain-enhancer of activated B Cells (NF-κB) and transforming growth factor-β2 (TGF-β2) pathways. This integration allows Lhx2 to sense both pro-inflammatory (NF-κB) and pro-fibrotic (TGF-β2) cues from the wound microenvironment, enabling a balanced response that maintains stem cell homeostasis while avoiding excessive inflammation or fibrosis (Mardaryev et al., 2011) (Figure 4).

Following injury, the dynamic expression pattern of *Lhx2* changes rapidly at the wound edge and in adjacent hair follicles. This spatiotemporal regulation ensures that re-epithelialization proceeds efficiently, while hair cycle activation is postponed until the epidermal barrier is restored. Consequently, Lhx2 coordinates tissue repair with long-term appendage function, achieving effective wound closure without compromising the regenerative capacity of the hair follicle niche (Mardaryev et al., 2011).

## 5 LHX2 dysregulation and diseases

Accumulating evidence indicates that dysregulation of LIM homeobox 2 (LHX2) is closely associated with a spectrum of human pathologies, including neurodevelopmental disorders, ocular malformations, liver fibrosis, osteoporosis, and various cancers. This section synthesizes these disease associations and highlights the underlying mechanistic links.

### 5.1 Non-neoplastic diseases

#### 5.1.1 Nervous system

In humans, LIM homeobox 2 (*LHX2*) haploinsufficiency causes intellectual disability, language impairment, microcephaly, and behavioral abnormalities, whereas complete loss of function leads to more severe phenotypes including optic cup arrest, anophthalmia, and forebrain malformations (Porter et al., 1997; Schmid et al., 2023). Region-specific defects have been well characterized. Agenesis of the corpus callosum arises from disrupted neuronal migration and aberrant glial wedge formation, highlighting the role of LHX2 in midline telencephalic development (Yang et al., 2024; Chinn et al., 2015). In the hippocampus, cooperative deficiency of *LHX2* and forkhead box G1 (*FOXG1*) causes medial cortical maldevelopment. Mechanistically, chromatin remodeling and cross-regulation with doublesex-and mab-3-related transcription factor A2 (DMRTA2), neurogenin 2 (NEUROG2), and paired box 6 (PAX6) impair the timing of neurogenesis and promote premature gliogenesis. Loss of microRNA (miR)-124a-mediated translational repression of *LHX2* exacerbates these defects, leading to abnormal neuronal aggregation and mossy fiber sprouting that further compromise cognitive function (Godbole et al., 2018; Suresh et al., 2023; Subramanian et al., 2011; Muralidharan et al., 2017b; Sanuki et al., 2011). In the thalamus, concomitant loss of *LHX2* and *LHX*9 blocks progenitor differentiation into mature thalamic neurons and permits ectopic expansion of Wnt-positive tissue, disrupting anteroposterior patterning and consequently interfering with sensory relay and consciousness regulation (Peukert et al., 2011). Pituitary development is also affected. *LHX2* deletion compromises infundibulum formation and alters anterior and intermediate lobe structure (Zhao et al., 2010). At the neural circuit level, dysregulation of guidance molecules such as slit guidance ligand 1 (SLIT1)/semaphorin 3 A (SEMA3A) and their receptors roundabout guidance receptor 1 (ROBO1)/ROBO2 disrupts the precise projection and topographic mapping of thalamocortical axons, further linking LHX2 dysfunction to circuit-level pathologies (Yang J. J. et al., 2020; Marcos-Mondéjar et al., 2012; Lakhina et al., 2007).

#### 5.1.2 Visual system

During early embryonic development, loss of *LHX2* leads to optic vesicle arrest, resulting in anophthalmia or microphthalmia, as the optic primordium fails to undergo proper evagination and patterning (Porter et al., 1997; Yun et al., 2009; Tétreault et al., 2009). In the retinal pigment epithelium (RPE), LHX2 synergizes with orthodenticle homeobox 2 (OTX2) to activate RPE-specific genes. Disruption of this interaction, particularly genetic variants affecting LHX2 binding sites, increases risk for age-related macular degeneration (Cohen-Gulkar et al., 2023). In the lens, LHX2 drives fiber cell differentiation by regulating neuroretina-derived fibroblast growth factors (FGFs). Loss of *LHX2* at this stage impairs lens fiber elongation and survival, leading to lens developmental abnormalities (Thein et al., 2016). Dysregulation of LHX2-mediated cell cycle exit in retinal progenitor cells indirectly affects lens morphogenesis, underscoring the interconnectedness of retinal and lens development (Thein et al., 2016; Gordon et al., 2013). In Müller glial cells, *LHX2* deficiency induces reactive gliosis, disrupting retinal ion and water homeostasis and contributing to the pathogenesis of glaucoma and diabetic retinopathy (De Melo et al., 2016a; De Melo et al., 2016b). LHX2 also maintains the balance between retinal neurogenesis and gliogenesis. Disruption of this balance promotes reactive gliosis and exacerbates retinal degenerative pathologies (De Melo et al., 2018). After optic nerve injury, LHX2 establishes a regenerative microenvironment by transcriptionally repressing semaphorin 3C (SEMA3C) (a chemorepellent for axons) and promoting retinal ganglion cell survival. Dysfunction of this process impairs axonal regeneration and delays functional recovery (Li et al., 2024).

#### 5.1.3 Digestive system

In *Lhx2* knockout mouse embryos, liver fibrosis develops spontaneously and progressively, without any additional injury stimulation. Histologically, the fibrosis is characterized by excessive deposition of type I and type III collagen, disorganized hepatic tissue architecture, and formation of abnormal tubular structures within the liver parenchyma (Porter et al., 1997; Kolterud et al., 2004b). *Lhx2* deficiency also causes liver hypoplasia and fatal embryonic anemia, further underscoring its irreplaceable role in maintaining liver homeostasis during development (Porter et al., 1997; Kolterud et al., 2004b). Given the critical role of Lhx2 in limiting hepatic stellate cell (HSC) activation, by keeping HSCs quiescent under physiological conditions, its loss of function directly upregulates profibrotic factors such as platelet-derived growth factor (PDGF), matrix metalloproteinases (MMPs), promotes extracellular matrix deposition, and disrupts the balance between fibrogenesis and matrix degradation (Wandzioch et al., 2004). Importantly, this mouse model recapitulates core pathological processes of human chronic liver disease, suggesting that restoring or enhancing Lhx2 activity in HSCs may reverse or delay fibrosis in patients (Wandzioch et al., 2004).

#### 5.1.4 Musculoskeletal system

Loss or downregulation of *LHX2* leads to bone loss and inflammatory bone destruction by relieving its inhibition of osteoclast differentiation, positioning LHX2 as a key endogenous suppressor of bone resorption (Kim et al., 2014; Takayanagi, 2007; Schett, 2009). Mechanistically, LHX2 binds directly to cellular Fos proto-oncogene (c-Fos) via its N-terminal LIM domains, preventing c-Fos from activating the master osteoclastogenic transcription factor nuclear factor of activated T Cells cytoplasmic 1 (NFATC1). Under normal physiological conditions, receptor activator of nuclear factor κ-B ligand (RANKL) signaling downregulates *LHX2* expression during the early stages of osteoclast differentiation, thereby releasing this brake and allowing physiological bone resorption to occur as needed for bone remodeling. When LHX2 function is lost or sufficiently reduced, excessive osteoclast generation occurs, leading to enhanced bone resorption that outpaces osteoblast-mediated bone formation, ultimately inducing osteoporosis (Kim et al., 2014). Pro-inflammatory cytokines such as tumor necrosis factor-α (TNF-α) downregulate *LHX2* expression, keeping it persistently low in the inflammatory microenvironment. This sustained downregulation attenuates LHX2-mediated inhibition of osteoclasts, resulting in abnormally active bone resorption that contributes to the pathogenesis of rheumatoid arthritis and other inflammatory bone diseases (Kim et al., 2014). Thus, restoring *LHX2* expression or pharmacologically targeting the LHX2/c-Fos axis represents a promising therapeutic strategy for treating osteoporosis and rheumatoid arthritis, potentially by re-establishing the physiological balance between bone resorption and formation.

### 5.2 Neoplastic diseases

LIM homeobox 2 (LHX2) acts as an oncogenic transcription factor that is aberrantly expressed across multiple human malignancies, driving proliferation, migration, invasion, metastasis, chemotherapy resistance, and cancer stem cell maintenance through diverse molecular mechanisms. Despite the diversity of cancer types, several common oncogenic pathways downstream of LHX2 recur across malignancies. These include: (i) phosphoinositide 3-kinase (PI3K)/protein kinase B (AKT)/mechanistic target of rapamycin (mTOR) axis activation (nasopharyngeal carcinoma, breast cancer, osteosarcoma); (ii) Wnt/β-catenin signaling stabilization (nasopharyngeal carcinoma, pancreatic ductal adenocarcinoma); (iii) microRNA (miRNA)-mediated LHX2 deregulation (lung, cervical, and osteosarcoma, via miR-124, miR-144, and miR-129–5p, respectively); and (iv) metabolic-epigenetic reprogramming (prostate cancer). The following sections detail these mechanisms within each anatomical system, highlighting both tissue-specific and shared features.

#### 5.2.1 Nervous system

In medulloblastoma (particularly Group 3 and Group 4 subtypes), LHX2 establishes a bone morphogenetic protein (BMP)-LHX2-activin A receptor type 1 (ACVR1) positive feedback loop that amplifies BMP signaling. BMP ligands induce SMAD family member 1 (SMAD1)/ SMAD5 phosphorylation and nuclear translocation, which transactivate *LHX2* expression. In turn, upregulated LHX2 binds the *Acvr1* promoter (encoding a BMP type I receptor) and enhances its expression, increasing tumor cell sensitivity to BMP signals. This self-reinforcing loop continuously amplifies BMP signaling, thereby enhancing cancer stem cell properties, self-renewal capacity, and chemotherapy resistance. Recurrent medulloblastomas often exhibit high LHX2 expression and enhanced BMP signaling. Knockdown of *LHX2* suppresses both BMP activity and tumor growth. Thus, targeting the LHX2-ACVR1 axis represents a potential therapeutic strategy for aggressive medulloblastoma (Ohata et al., 2025).

#### 5.2.2 Respiratory system

In nasopharyngeal carcinoma, LHX2 directly transactivates fibroblast growth factor 1 (FGF1), leading to fibroblast growth factor receptor (FGFR) activation and downstream PI3K/AKT signaling. This inactivates glycogen synthase kinase-3β (GSK-3β), allowing β-catenin to stabilize and translocate to the nucleus. In the nucleus, β-catenin drives transcription of epithelial-mesenchymal transition (EMT) master regulators zinc finger E-box binding homeobox 1 (ZEB1) and twist family bHLH transcription factor 1 (*TWIST1*), thereby promoting tumor cell migration, invasion, and distant metastasis. Targeting this axis with FGFR inhibitors (e.g., AZD4547) or *FGF1* small interfering RNA (siRNA) effectively suppresses tumor growth, offering a potential therapeutic strategy for patients with high *LHX2* expression (Xie et al., 2022).

In non-small cell lung cancer, the regulatory mechanism is distinct. miR-124 expression is frequently downregulated and inversely correlates with LHX2 levels. miR-124 suppresses *LHX2* by binding to its 3′-untranslated region (3′-UTR). Overexpression of miR-124 or silencing of *LHX2* significantly reduces migration and invasion, whereas knockdown of miR-124 produces the opposite effect. These findings confirm the functional relevance of this regulatory axis in lung cancer (Yang et al., 2017).

#### 5.2.3 Digestive system

In pancreatic ductal adenocarcinoma, *LHX2* is specifically overexpressed and acts as a molecular bridge, facilitating T-cell factor 4 (TCF4) binding to β-catenin to form a stable LHX2/TCF4/β-catenin ternary transcriptional complex. This complex markedly enhances the transactivation of downstream oncogenic target genes, thereby continuously driving tumor cell proliferation and tumor growth. Disrupting the LHX2-β-catenin interaction partially abrogates the pro-tumorigenic activity of LHX2, underscoring the essential role of this complex (Zhou et al., 2014).

In esophageal squamous cell carcinoma, *LHX2* is also significantly upregulated. It directly binds to the promoter region of the downstream target gene serine protease inhibitor E2 (*SERPINE2*) and transcriptionally activates its expression. SERPINE2, as a key functional mediator of LHX2-driven malignancy, promotes tumor cell proliferation, migration, invasion, and metastasis. Rescue experiments further demonstrate that restoring *SERPINE2* expression completely reverses the tumor-suppressive effect of *LHX2* knockdown, indicating that the LHX2-SERPINE2 axis is an important oncogenic pathway in esophageal squamous cell carcinoma (Li et al., 2022b).

#### 5.2.4 Reproductive system

In neuroendocrine prostate cancer (an aggressive subtype emerging after long-term androgen deprivation therapy), LHX2 drives a metabolic-epigenetic positive feedback loop. Androgen receptor inhibition (e.g., with enzalutamide) induces metabolic reprogramming with enhanced glycolysis and abundant lactate production. Lactate triggers histone H3 lysine 18 lactylation (H3K18la), which epigenetically activates *LHX2* transcription. Activated LHX2 upregulates lactate dehydrogenase A (LDHA), further accelerating lactate generation and closing a self-amplifying loop. Concurrently, LHX2 activates DNA methyltransferase 1 (DNMT1), leading to genome-wide DNA methylation remodeling and driving neuroendocrine transdifferentiation (upregulating delta-like ligand 3 (DLL3) and neuron-specific enolase (NSE)). The antiviral drug paritaprevir directly binds and inhibits LHX2, blocking this loop and suppressing tumor growth (Jiang et al., 2025).

In cervical cancer, the mechanism is entirely different. *LHX2* is a direct target of miR-144. LHX2 is elevated in cisplatin-resistant cells, where it enhances resistance by inhibiting apoptosis and promoting survival. miR-144 suppresses *LHX2* by binding to its 3′-UTR. Downregulation of miR-144 (e.g., upon cisplatin treatment) relieves this inhibition, upregulating *LHX2* and driving resistance. Restoring miR-144 reduces LHX2 levels, partially reverses cisplatin resistance, and induces apoptosis (Shi et al., 2019).

#### 5.2.5 Musculoskeletal system

In osteosarcoma, *LHX2* is significantly upregulated. miR-129–5p is frequently downregulated and directly suppresses *LHX2* by binding to its 3′-UTR, thereby inhibiting *LHX2* translation. Conversely, *LHX2* overexpression promotes malignancy and inhibits autophagy via the mTOR pathway. Specifically, LHX2 activates mTOR, which suppresses autophagic flux, leading to increased proliferation, migration, and invasion. Silencing *LHX2* reduces these malignant phenotypes and induces autophagic cell death, as reflected by increased autophagic markers and reduced viability. Thus, the miR-129–5p/LHX2/mTOR axis represents a critical regulatory network in osteosarcoma progression and a potential therapeutic target (Song et al., 2019).

#### 5.2.6 Hematopoietic system

In chronic myeloid leukemia (CML), *LHX2* is aberrantly expressed in hematopoietic cells (normally absent in healthy counterparts) and promotes proliferation through a cell-nonautonomous mechanism. This mechanism depends on KIT ligand (KITLG) and autocrine signals that remodel the bone marrow niche to favor malignant cell expansion. Specifically, *LHX2*-expressing cells enhance production of niche factors supporting hematopoietic stem/progenitor cell survival and self-renewal, thereby creating a permissive microenvironment for leukemic progression (Pinto Do et al., 2002; Richter et al., 2003; Wu et al., 1996).

Transplantation of *Lhx2*-expressing cells into recipient mice induces a chronic myeloproliferative disorder resembling human CML. This disorder can progress from chronic phase to acute leukemia, faithfully recapitulating the chronic-to-blast evolution. Compared with the classic breakpoint cluster region-Abelson (BCR-ABL) oncogene, Lhx2 induces a more indolent, cytokine-dependent disease, yet both share features of leukocytosis and progression potential. These observations suggest that Lhx2 and BCR-ABL may operate through independent or partially overlapping pathogenic pathways. Thus, LHX2 provides a unique model for studying CML pathogenesis and hematopoietic stem cell pathology, as well as a potential therapeutic target (Pinto Do et al., 2002; Richter et al., 2003; Wu et al., 1996).

#### 5.2.7 Mammary gland

In breast cancer, *LHX2* is significantly upregulated and acts as a key oncogenic transcription factor. It directly activates the PI3K/AKT/mTOR signaling pathway, thereby enhancing tumor cell proliferation, migration, invasion, and colony formation while inhibiting apoptosis and conferring a marked survival advantage. Clinically, high *LHX2* expression correlates with poor patient prognosis and aggressive tumor features. Moreover, breast cancer tissues with high *LHX2* expression exhibit significantly increased infiltration of T helper 1 (Th1) and Th2 cells, as well as enrichment of T-cell activation-related signaling pathways. This suggests that LHX2 may indirectly influence tumor progression by remodeling the tumor immune microenvironment, in addition to its cell-autonomous oncogenic effects (Zhang et al., 2023).

## 6 Discussion and future perspectives

LIM homeobox 2 (LHX2) exhibits three distinct functional modes across different biological contexts. During embryogenesis, it acts as a selector gene and lineage determinant, directing regional specification and cell fate commitment in the cerebral cortex, hippocampus, and retina (Mangale et al., 2008; Chou et al., 2009; Hägglund et al., 2011). In adult tissues, it functions as a key negative regulator, keeping astrocytes, hepatic stellate cells (HSCs), and osteoclast precursors quiescent by actively suppressing activation-associated gene programs (Wandzioch et al., 2004; Kim et al., 2014; Iyer et al., 2025). Upon injury, LHX2 switches to a pro-regenerative mode, promoting re-epithelialization, axon regrowth, and niche remodeling (Mardaryev et al., 2011; Li et al., 2024). Nevertheless, several critical knowledge gaps impede a comprehensive understanding of LHX2 biology.

Whether the phase separation mechanism observed in olfactory receptor choice operates in other tissues, such as the cerebral cortex or hippocampus, and whether it is cell-type-specific or modulated by post-translational modifications remain open questions. Phase separation can concentrate transcription factors and cofactors into dynamic condensates, but the rules governing condensate formation, for example, the role of intrinsically disordered regions and partner proteins, remain largely unexplored for LHX2. Elucidating these principles will determine whether phase separation is a general mechanism for LHX2-mediated regulation or a specialized adaptation for olfactory receptor choice.

The evolutionary basis of species-specific functional reversal also requires investigation. LHX2 expands murine hematopoietic stem cells but suppresses human induced pluripotent stem cell (iPSC)-derived counterparts. This inhibition can be overcome by engineering an enhanced transcriptional activation domain (Kitajima et al., 2024). This striking divergence suggests that the transcriptional activation potential of LHX2 has been repurposed during evolution, possibly through changes in its interacting cofactors or chromatin landscape. Whether similar species-specific differences exist in other organ systems, such as in neural development or liver homeostasis, awaits comparative genomics and structure-function analyses. Systematic mapping of *LHX2* cistromes and protein interactomes across species will help decipher the molecular underpinnings of this functional reversal and guide the design of human-compatible *LHX2* variants.

The precise spatiotemporal control of LHX2 dosage and activity is also poorly understood. *LHX2* expression is fine-tuned by microRNA (miR)-124a (Sanuki et al., 2011), and its aberrant elevation causes circuit defects. The strict temporal window in cortical development illustrates tight constraints: ablation before embryonic day (E) 10.5 causes a fate switch, whereas ablation 1 day later does not (Chou et al., 2009). These observations imply that even modest fluctuations in LHX2 levels or timing can profoundly alter developmental outcomes. Moreover, because *LHX2* is expressed in multiple organs, enhancing its function for optic nerve regeneration might inadvertently affect hair follicle cycling or HSC quiescence (Li et al., 2024). Unraveling how LHX2 dosage integrates with cell-type-specific chromatin states and signaling environments will be critical for predicting the consequences of therapeutic modulation.

Addressing these gaps will require integrated approaches that combine molecular engineering, single-cell technologies, and comparative biology. For example, engineering humanized *LHX2* variants could bypass species barriers for *ex vivo* expansion of human HSCs (Kitajima et al., 2024). In addition, single-cell trajectory analysis combined with temporal lineage tracing would reveal how LHX2 dosage biases progenitor fate in a context-dependent manner. Context-specific delivery using Adeno-Associated Virus (AAV) vectors with cell-type-specific promoters could achieve tissue-restricted intervention, mitigating off-target effects. Furthermore, visualizing phase separation *in vivo* via clustered regularly interspaced short palindromic repeats (CRISPR) knock-in of fluorescent tags would test whether LHX2 assembles transcriptional hubs in the developing cortex or retina. Finally, cross-species comparisons of LHX2 cistromes and interactomes will illuminate the molecular basis of functional divergence and inform the rational design of LHX2-based therapeutics. Together, these efforts will pave the way for targeting LHX2 in human disease.

## 7 Conclusion

LHX2 operates as a triphasic regulator: a selector gene in development, a quiescence factor in homeostasis, and a pro-regenerative switch after injury. Its dysregulation underlies neurodevelopmental disorders, liver fibrosis, osteoporosis, and multiple cancers. Spatiotemporally precise modulation of LHX2 activity holds therapeutic promise for regenerative medicine and genetic diseases.

## Glossary

3′-UTR: 3′-untranslated region
α-SMA: α-smooth muscle actin
AAV: Adeno-associated virus
ACVR1: Activin a receptor type 1
AER: Apical ectodermal ridg
AKT: Protein kinase B
AMD: Age-related macular degeneration
BAF: BRG1/BRM-associated factor
BCR-ABL: Breakpoint cluster region-abelson
Best1: Bestrophin 1
BMP: Bone morphogenetic protein
CBP: CREB-binding protein
c-Fos: Cellular fos proto-oncogene
cDNA: Complementary DNA
CML: Chronic myeloid leukemia
CRISPR: Clustered regularly interspaced short palindromic Repeats
CUX2: Cut-like homeobox 2
DLL3: Delta-like ligand 3
DMRTA2: Doublesex and mab-3-related transcription factor A2
DNMT1: DNA methyltransferase 1
E: Embryonic day
EBF: Early B-cell factor
EMT: Epithelial-mesenchymal transition
ETS: E26 transformation-specific
FEZF2: FEZ family zinc finger 2
FGF: Fibroblast growth factor
FGF1: Fibroblast growth factor 1
FGFR: Fibroblast growth factor receptor
FOXG1: Forkhead box G1
GFAP: Glial fibrillary acidic protein
GnRH: Gonadotropin-releasing hormone
GSK-3β: Glycogen synthase kinase-3β
H3K18la: Histone H3 lysine 18 lactylation
H3K9me2: Histone H3 lysine 9 dimethylation
HD: Homeodomain
HDAC2: Histone deacetylase 2
HES1: hes family bHLH transcription factor 1
HES5: hes family bHLH transcription factor 5
Hgf: Hepatocyte growth factor
HSC: Hepatic stellate cell
HSPC: Hematopoietic stem/progenitor cell
iPSC: Induced pluripotent stem cell
KITLG: KIT ligand
LADLRM: LHX2-associated distal limb regulatory module
LASARM: LHX2-associated sub-AER regulatory module
LDB1: LIM domain binding protein 1
LDHA: Lactate dehydrogenase A
LEF: Lymphoid enhancer factor
Lgr5: Leucine-rich repeat-containing G-protein coupled Receptor 5
LHX2: LIM homeobox 2
LMX1: LIM homeobox transcription factor 1
LOT: Lateral olfactory tract
LSD1: Lysine-specific demethylase 1
MAPK: Mitogen-activated protein kinase
miR: microRNA
miRNA: microRNA
MMPs: Matrix metalloproteinases
MRG1: MSG1-related gene 1
mRNA: Messenger RNA
mTOR: Mechanistic target of rapamycin
NEUROG2: Neurogenin 2
NF-κB: Nuclear factor κ-light-chain-enhancer of activated B Cells
NFATC1: Nuclear factor of activated T Cells cytoplasmic 1
Notch: Neurogenic locus notch homolog protein
NSE: Neuron-specific enolase
OR: Odorant receptor
OSN: Olfactory sensory neuron
OTX2: Orthodenticle homeobox 2
p300: E1A binding protein p300
PAX6: Paired box 6
PDGF: Platelet-derived growth factor
PI3K: Phosphoinositide 3-kinase
PROX1: Prospero homeobox 1
RANKL: Receptor activator of nuclear factor κ-B ligand
Ras: Rat sarcoma
RAX: Retina and anterior neural fold homeobox
RBBP4: Retinoblastoma-binding protein 4
RGC: Retinal ganglion cell
ROBO1: Roundabout guidance receptor 1
ROBO2: Roundabout guidance receptor 2
RPC: Retinal progenitor cell
RPE: Retinal pigment epithelium
RPE65: RPE-specific 65 kDa
SEMA3A: Semaphorin 3A
SEMA3C: Semaphorin 3C
SEMA6A: Semaphorin 6A
SERPINE2: Serine protease inhibitor E2
SHH: Sonic hedgehog
siRNA: Small interfering RNA
SIX3: SIX homeobox 3
SLIT1: Slit guidance ligand 1
SMAD1: SMAD family member 1
SMAD5: SMAD family member 5
SMAD6: SMAD family member 6
SOX8: SRY-box transcription factor 8
SOX9: SRY-box transcription factor 9
SOX11: SRY-box transcription factor 11
TBP: TATA-box binding protein
TCF: T-cell factor
TCF4: T-cell factor 4
TGF-β: Transforming growth factor-β
Th1: T Helper 1 (cell)
Th2: T Helper 2 (cell)
TNF-α: Tumor necrosis factor-α
TRPM1: Transient receptor potential cation channel subfamily M Member 1
TWIST1: Twist family bHLH transcription factor 1
Wnt: Wingless/Int-1
WNT3A: Wnt family member 3A
ZEB1: Zinc finger E-box binding homeobox 1
ZPA: Zone of polarizing activity.
